# Robotic body weight support enables safe stair negotiation in compliance with basic locomotor principles

**DOI:** 10.1186/s12984-019-0631-8

**Published:** 2019-12-23

**Authors:** M. Bannwart, E. Rohland, C. A. Easthope, G. Rauter, M. Bolliger

**Affiliations:** 1 Spinal Cord Injury Center, Balgrist University Hospital, Forchstrasse 340, CH-8008 Zurich, Switzerland; 2 Sensory Motor Systems Lab, Department of Health Sciences and Technology, Swiss Federal Institute of Technology, Zurich, Switzerland; 3 BIROMED-Lab, Department of Biomedical Engineering, University Basel, Gewerbestrasse 14, CH-4123 Basel, Allschwil Switzerland; 4 Cereneo Center for Interdisciplinary Research, Vitznau, Switzerland

**Keywords:** Body weight support, Gait pattern, Myoelectric activity, Stair negotiation, Unloading

## Abstract

**Background:**

After a neurological injury, mobility focused rehabilitation programs intensively train walking on treadmills or overground. However, after discharge, quite a few patients are not able to independently negotiate stairs, a real-world task with high physical and psychological demands and a high injury risk. To decrease fall risk and improve patients’ capacity to navigate typical environments, early stair negotiation training can help restore competence and confidence in safe stair negotiation. One way to enable early training in a safe and permissive environment is to unload the patient with a body weight support system. We here investigated if unloaded stair negotiation complies with basic locomotor principles, in terms of enabling performance of a physiological movement pattern with minimal compensation.

**Methods:**

Seventeen able-bodied participants were unloaded with 0–50% bodyweight during self-paced ascent and descent of a 4-tread staircase. Spatio-temporal parameters, joint ranges of motion, ground reaction forces and myoelectric activity in the main lower limb muscles of participants were compared between unloading levels. Likelihood ratio tests of separated linear mixed models of the investigated outcomes assessed if unloading affects the parameters in general. Subsequent post-hoc testing revealed which levels of unloading differed from unsupported stair negotiation.

**Results:**

Unloading affected walking velocity, joint ranges of motion, vertical ground reaction force parameters and myoelectric activity in all investigated muscles for stair ascent and descent while step width and single support duration were only affected during ascent. A reduction with increasing levels of body weight support was seen in walking velocity (0.07–0.12 m/s), ranges of motion of the knee and hip (2–10°), vertical ground reaction force peaks (10–70%) and myoelectric activity (17–70%). An increase with unloading was only seen during ascent for ankle range of motion and tibialis anterior activity at substantial unloading.

**Conclusions:**

Body weight support facilitates stair negotiation by providing safety and support against gravity. Although unloading effects are present in most parameters, up to 30% body weight support these changes are small, and no dysfunctional patterns are introduced. Body weight support therefore fulfills all the necessary requirements for early stair negotiation training.

## Background

Injuries to the central nervous system result in a wide range of disabilities of which more than 60% show gait dysfunctions [1]. As a consequence, these patients often demonstrate slow or abnormal gait and impaired balance which result in a greatly increased risk of falling with high probability of severe secondary injuries [2]. At an advanced stage, gait dysfunctions and fear of falling can lead to a loss of independence, social isolation and mobility restrictions [2] - factors strongly related to a decreased quality of life [3]. Therefore, a large proportion of modern rehabilitation programs focus on gait and balance training in compliance with locomotor training principles. These principles are known to maximize recovery and restoration and state that weight-bearing through legs should be maximized, appropriate sensory cues and task-specific, physiological kinematics need to be provided while compensatory strategies should be minimized [4]. But locomotor training should not only focus on simple walking or balance, but also on advanced activities like curb and stair negotiation which are similarly indispensable for independent living. Paolucci et al. however report that of initially non-ambulatory patients with stroke, only 4.58% regain the ability to independently negotiate stairs while 50.57% regain the ability to walk [5]. One reason behind this is that negotiating stairs is much more challenging than overground walking [6]. The greater complexity of stair negotiation and the increased risk of falling compared to level ground walking originates from higher physical demands such as the need for i) larger joint ranges of motion (ROMs), ii) higher muscular strength, iii) better cardiovascular fitness [7], iv) more precise foot placement which relies on accurate visual feedback [8] and increased stability [9]. In addition, stair negotiation is psychologically challenging due to the increased probability of serious injury in case of a fall compared to walking on level ground. To restore a high level of independence, it is desirable to boost patients’ capabilities and confidence in safe stair negotiation. Optimally, patients would start stair negotiation training early in their rehabilitation process to maximally benefit from the optimal time window during which the central nervous system might show increased neuroplasticity [10, 11]. Appropriate assistance and security are a requirement for early stair climbing training, however this puts a large burden on therapists in terms of support forces. One way to provide large supportive forces is via robotic devices. Robotic rehabilitation technology that assists training of stair negotiation from an early time point on is however rare and limited to few devices such as end-effector-based gait trainers, ceiling-mounted BWS systems, and wearable exoskeletons [12–17]. Compared to gait trainers, BWS systems and wearable exoskeletons have the advantage that they allow training of real stair walking which helps provide the appropriate afferent sensory input to relearn the task. Wearable exoskeletons, the most recently emerged of these technologies, are however still struggling with fall safety mechanisms and require users to rely on crutches for balancing resulting in compensatory arm activity [18]. BWS systems on the other hand do not seem to substantially hinder or compromise physiological movement execution which was at least shown for able-bodied and patients with incomplete spinal cord injury during overground walking with up to 30% of BWS [19–21]. By changing BWS, the intensity of the training can be adapted to the individual patient and his/her capabilities. Ceiling-mounted BWS systems can therefore be a promising tool to support stair negotiation in patients with remaining voluntary muscle control. However, the effect of BWS on movement performance specifically during stair negotiation has to our best knowledge not yet been investigated. It is therefore not clear if BWS hinders physiological performance of stair ambulation, something which must be first investigated in an able-bodied population.

Therefore, this paper aims at providing insights into effects of different levels of BWS on the biomechanics and myoelectric activity during stair negotiation. We used the FLOAT (The FLOAT, RehaStim Medtech AG, Germany) BWS system for our investigations. FLOAT can apply different levels of unloading as well as horizontal assistance forces during a broad range of training tasks including ground level walking, standing up/sitting down, as well as stair negotiation [15, 20–26]. From previous investigations of the FLOAT and other BWS systems during overground walking in able-bodied subjects, it is known that with higher levels of BWS temporal parameters change towards shorter stance durations and lower limb joint ROMs are reduced apart from inconclusive evidence for the ankle [19, 20]. Kinetics and myoelectric activity show in most cases reductions with some inconclusive evidence regarding compensatory activity. The general consensus is however that deviations from physiological movement patterns are small and negligible up to 30% BWS [19, 20]. A similar understanding of alterations introduced by BWS in able-bodied individuals during stair negotiation is important for validating the task-specificity of BWS stair training, which optimally transfers to daily life [27]. We hypothesize that BWS, does not induce large deviations in lower limb kinematic patterns while reducing neuromuscular demand without introducing compensatory activity. If this holds true, BWS stair training should be safe to apply for physiological training of stair negotiation in patients with neurological diseases.

## Methods

### Participants

We included 17 able-bodied volunteers (9~female and 8~male) in this study. All volunteers provided their written informed consent prior to participation. The study was approved by the local ethics committee of the Canton of Zurich (KEK Nr. PB_2016–0193) and conducted in accordance with the Declaration of Helsinki.

### Equipment

**Fig. 1:**
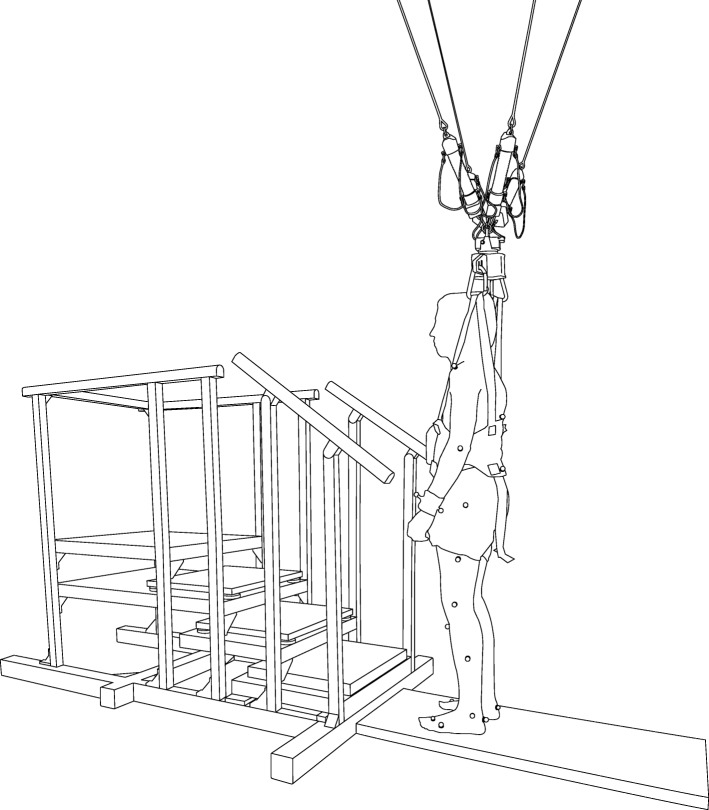
Experimental setup. Stair negotiation setup with the 4-step custom-made staircase with two force plates and the FLOAT BWS system attached to an individual with harness. The individual is equipped with reflective markers

To investigate the effects of unloading on stair negotiation performance, we used a custom-made staircase (Fig. 1). This consisted of a frame together with handrails made of aluminum profiles (Bosch Rexroth AG, Lohr am Main, Germany) and had four treads including the top platform. Stair dimensions were chosen to adhere to common stair norms (Norm SIA 500 SN 521500) with a riser of 0.175 m and a tread depth of 0.3 m. Stair width was chosen to be 0.7 m which is a comfortable width for walking up and down for one person allowing to grasp the handrails at both sides simultaneously.

Participants were unloaded during the stair negotiation task using the FLOAT BWS system. The FLOAT is a tendon-based parallel robot which allows practically unrestricted movement in a large, room architecture-dependent workspace volume (2.35 m wide × 7.82 m long × 3.5 m high for our setup). Users are connected to the FLOAT via a harness. Through the harness, the FLOAT provides these users with up to 60% BWS and 10% horizontal forces (% BW) and catches them in case of falls. A description of the basic mechanical working principle of the first prototype version of FLOAT and the current systems’ transparency evaluation can be found elsewhere [15, 26].

Kinematic, kinetic and EMG data were recorded for different levels of BWS unloading. A motion capture system (Vicon Motion Systems Ltd., Oxford, UK) together with passive, reflective markers to sample kinematic data at 200 Hz. A wireless EMG system (Aktos Nano, Myon AG, Schwarzenberg, Switzerland) provided EMG data at 1000 Hz that were hardware-filtered with a first order bandpass filter (10–500 Hz). EMG surface electrodes were bilaterally placed on the following lower limb muscles (according to SENIAM guidelines): gluteus maximus (GMax), rectus femoris (RF), biceps femoris (BF), vastus lateralis (VL), gastrocnemius medialis (GM), and tibialis anterior (TA). The second and third steps (i.e. the middle steps) were each equipped with a force plate (9260AA, Kistler Group, Winterthur, Switzerland) for acquiring ground reaction forces (GRFs) at 1 kHz.

### Experimental protocol

**Fig. 2:**
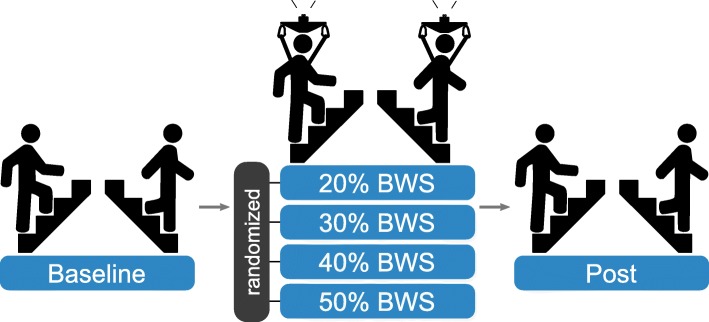
Experimental protocol. Order and randomization of experimental conditions

To assess the effect of different levels of unloading on stair negotiation performance, we compared the following 6 conditions: no unloading (baseline & post), 20% BWS, 30% BWS, 40% BWS and 50% BWS. Participants were weighed with a scale and wore the same harness during all conditions including baseline and post. All measurements started with a no unloading condition (baseline) followed by one of the four unloading conditions in randomized order and concluded the protocol with a second no unloading measurement (post) to test for possible fatigue or adaptation effects (Fig. 2). Before each condition, subjects walked up and down the stairs at least twice until they self-reported feeling comfortable and accustomed to the unloading force. This enabled familiarization while also providing a washout period to decrease potential carry-over effects.

Subjects performed at least 7 ascending and 7 descending trials of each condition at a self-selected speed, always freely choosing their initiation limb. Data was recorded from the starting position at top or bottom of stairs until the task was completed. Ascents and descents were recorded alternatingly with short breaks of 10–15 s in between starting on top of the stairs or on the ground. For descents, fall detection sensitivity was reduced at trial start for a 10-s interval to prevent an incorrect detection of the participants downward motion as a fall.

### Data processing

Synchronously recorded kinematic, kinetic and EMG data were post-processed offline for further analysis. Kinematic data was processed with Vicon Nexus Software (Vicon Nexus, Version 2.6.0). The Plug-in Gait model was used for marker labeling, gaps in the trajectories were filled with appropriate gap-filling algorithms provided by Vicon Nexus. Data was filtered with a Woltring filter with a tracking volume-specific mean-square error value of 15 mm^2^. Foot strike and foot off events were set based on force plate data and a threshold of 20 N. Further processing (MATLAB R2019a, The Mathworks Inc., Natick, USA) included identification of gait events with no available force plate data and segmentation of continuous data into individual gait cycles (from foot strike to foot strike). Stance and swing phases were normalized to the mean percentage of all stance and swing phases.

Anteroposterior, mediolateral, and vertical GRF data from the force plates was filtered with a recursive fourth order low-pass Butterworth filter with a cut-off frequency of 20 Hz. Force values were normalized to participants’ BW and a threshold of 3% was used to find start and end of ground contact and to segment data into single strides [28].

Raw EMG data was visually inspected for motion artefacts (e.g. harness-sensor interactions) and data with clear-cut artifacts (296 of 11,424 gait cycles) were removed from analysis. Noise in the remaining raw signals was removed with a 20–450 Hz recursive fourth order Butterworth bandpass filter [29]. For plotting continuous EMG activity, the denoised signals were rectified and smoothed with a recursive second order low-pass filter and a cut-off frequency of 5 Hz to extract the envelopes of the signals [30]. For each subject, the EMG envelopes were segmented into single strides and normalized to the mean of the upper 5% of all baseline trials to be comparable between the same muscles across subjects.

### Outcome metrics

A range of frequently reported spatio-temporal, kinematic, kinetic and myoelectric parameters was selected to investigate the effect of unloading on these different domains and compare them with normative data from previous studies [7, 31–33].

#### Spatio-temporal

Parameters calculated from the processed data were stride length, step length and step width (from marker positions at foot strike), stance and swing phase durations (% of gait cycle), single and double support phases (% of gait cycle) and center of mass velocity (meter per second).

#### Kinematics

Parameters included sagittal ankle, knee and hip joint ROMs. These were obtained using the Vicon Nexus Plug-In Gait full body model and subject specific anthropometric measurements taken during subject preparations.

#### Kinetics

Parameters extracted from steps on force plates were force peaks (Fz2, Fz4) and plateaus (Fz3) from vertical GRFs. The extraction followed a routine described by Stüssi and Debrunner [34].

#### Myoelectric activity

For statistical comparisons of myoelectric activity, root mean square (RMS) values over stance and swing phases were calculated from the denoised EMG signals. For each subject, the RMS values were normalized by the median stance or swing RMS value over each subject’s baseline trials. The median was chosen over the mean to reduce distortion by outliers.

### Statistical analysis

#### Model description

All statistical procedures were conducted with the R statistical computing environment (v3.6.1, R Core Team, 2018) using R Studio (v1.2.1335, RStudio Team, 2016) as interface and the lmerTest (v3.0–1, [35]), lme4 (v1.1–21, [36]), and multcomp (v1.4–10, [37]) packages. The lmerTest package was used to create multiple, univariate linear mixed effects models (LMMs) for each of the selected parameters of the chosen outcome metrics (dependent variables). The analysis only included gait cycles from the middle stair steps to reduce transition effects [38]. For each parameter, two separate models were built specifically for ascending and descending directions. All the built models were random intercept models with subjects as random effect and unloading (categorical factor with 6 levels corresponding to baseline, 20% BWS, 30% BWS, 40% BWS, 50% BWS and, post condition), sex (male and female) and, body mass index (BMI) as fixed effects. We report here the R-typical notation for a generalized version of these models:

$$ {\mathrm{outcome}}_{\mathrm{direction}}\sim \mathrm{unloading}+\mathrm{sex}+\mathrm{BMI}+\left(1|\mathrm{subject}\right). $$

BMI was included to avoid collinearity issues which could result from having multiple, highly correlated factors like subject height and weight and was mean centered to obtain intercept estimates for mean BMI values instead of zero. For myoelectric data, “gait phase” was added as additional fixed effect to investigate stance and swing phase effects. We also included an “unloading * gait phase” interaction term into the LMMs containing myoelectric outcome parameters if unloading was a significant fixed effect:

$$ {\mathrm{outcome}}_{\mathrm{direction}}\sim \mathrm{unloading}\ast \mathrm{gait}\ \mathrm{phase}+\mathrm{sex}+\mathrm{BMI}+\left(1|\mathrm{subject}\right). $$

This way, different unloading effects on stance and swing phase can be quantified. If the interaction itself did not turn out significant, only fixed effects without interaction terms were kept.

#### Model Verification & Reporting

Model assumptions were tested with Q-Q plots and plots of residuals against fitted values to detect deviations from linearity, homoscedasticity and normality. In case of deviations we transformed the dependent variables to improve model fitting. We report *p*-values from chi-square-based likelihood ratio tests (LRT) of each model with the main fixed effect unloading against the model without unloading. LRT tests compare the likelihood of seeing the observed data given the model with unloading versus the model without unloading as a fixed effect and can therefore tell us if unloading significantly explains the observed data. The significance level α was set to 0.05 and p-values were adjusted for all model comparisons using Holm-Bonferroni correction implemented in the multcomp package. For post-hoc tests, we used Dunnett’s Test to compare the baseline level (estimated LMM intercept) against all unloading levels (estimated LMM mean differences to the intercept) for all models with a main effect of unloading. Hence, reported results include estimates of model intercepts and mean differences with standard errors (which are assumed to be homogenous over a single fixed factor if datasets are balanced) of all fixed effects and an identifier for significant post-hoc tests (see Additional file 1 for detailed post-hoc test statistics). For EMG models with an interaction, a superfactor between unloading and gait phase was created to allow comparable post-hoc testing with contrasts specified between stance baseline and all stance unloading levels as well as swing baseline and all swing unloading levels.

## Results

### Population

Participants had a mean age of 34.24 ± 15.41 years, mean height of 1.71 ± 0.09 m and mean weight of 71.18 ± 13.38 kg (mean ± 1SD).

### Spatio-temporal parameters

#### Ascent

Parameters significantly affected by unloading were velocity (χ^2^(5) = 117.55, *p* = 2.59e-22), step width (χ^2^(5) = 30.41, *p* = 1.47e-4), and single support duration (χ^2^(5) = 16.87, *p* = 4.27e-2, see Additional file 1 for non-significant chi-square test statistics). Post-hoc comparisons of mean differences between baseline and unloading conditions show that for velocity and single support duration all unloading levels are significantly different from baseline apart from the post measurement (Table 1). Velocity is reduced, while single support duration is increased. Step width on the other hand shows a reduction only at 20 and 30% BWS.

**Table 1:** LMM mean difference estimates for various gait parameters

	Intercept (Base)	ΔBWS20	ΔBWS30	ΔBWS40	ΔBWS50	ΔPost	Δmale	ΔBMI
Ascent								
Velocity (m/s)	0.38 (0.02)	**−0.07 (0.01)**	**− 0.07 (0.01)**	**− 0.09 (0.01)**	**−0.11 (0.01)**	− 0.01 (0.01)	0.03 (0.02)	0.00 (0.00)
Step Width (m)	0.064 (0.008)	**−0.022 (0.005)**	**−0.023 (0.005)**	− 0.007 (0.005)	−0.004 (0.005)	− 0.008 (0.005)	−0.01 (0.01)	0.00 (0.00)
Sing. Supp. (%)	36.8 (0.9)	**1.5 (0.5)**	**1.3 (0.5)**	**1.6 (0.5)**	**1.7 (0.5)**	0.6 (0.5)	0.01 (0.01)	0.00 (0.00)
Hip ROM (°)	58.39 (1.40)	**−2.87 (1.05)**	**−3.68 (1.05)**	**− 5.71 (1.05)**	**−8.09 (1.05)**	−0.27 (1.05)	− 2.38 (1.86)	− 0.42 (0.36)
Knee ROM (°)	83.70 (3.33)	**− 3.05 (1.18)**	**−4.60 (1.18)**	**−8.84 (1.18)**	**− 11.43 (1.18)**	−0.37 (1.18)	2.73 (4.95)	−0.93 (0.96)
Ankle ROM (°)	47.47 (1.89)	**7.69 (1.03)**	**7.78 (1.03)**	**9.71 (1.03)**	**9.36 (1.03)**	1.36 (1.03)	−8.38 (2.69)	−0.73 (0.52)
Fz2 (% BW)	104.78 (1.74)	**−10.43 (1.11)**	**−20.54 (1.11)**	**−31.70 (1.11)**	**−43.13 (1.11)**	−0.60 (1.11)	2.70 (2.41)	−0.63 (0.47)
Fz3 (% BW)	80.75 (1.46)	**−4.29 (1.40)**	**−16.55 (1.40)**	**−27.21 (1.40)**	**−37.58 (1.41)**	3.19 (1.41)	−5.82 (1.75)	−0.16 (0.34)
Fz4 (% BW)	113.50 (1.55)	**−28.52 (1.49)**	**−37.90 (1.49)**	**−48.33 (1.49)**	**−59.54 (1.49)**	−1.47 (1.49)	−0.53 (1.85)	− 0.63 (0.36)
Descent								
Velocity (m/s)	0.42 (0.02)	**−0.08 (0.01)**	**−0.09 (0.01)**	**− 0.11 (0.01)**	**−0.12 (0.01)**	− 0.01 (0.01)	0.04 (0.03)	0.00 (0.01)
Hip ROM (°)	26.89 (0.93)	**−1.72 (0.54)**	**−1.76 (0.54)**	**−3.02 (0.54)**	**−4.49 (0.54)**	−0.17 (0.54)	− 1.43 (1.32)	− 0.55 (0.26)
Knee ROM (°)	87.86 (1.92)	**−3.00 (0.82)**	**− 3.73 (0.82)**	**−4.12 (0.82)**	**−5.91 (0.82)**	− 1.24 (0.82)	−2.12 (2.81)	− 0.70 (0.55)
Ankle ROM (°)	65.94 (1.99)	−1.45 (0.81)	**−4.03 (0.81)**	**−5.40 (0.81)**	**−8.28 (0.81)**	1.14 (0.81)	−9.95 (2.93)	−0.25 (0.57)
Fz2 (% BW)	126.48 (2.67)	**−37.58 (2.09)**	**−46.33 (2.09)**	**−59.40 (2.09)**	**−72.49 (2.09)**	−1.04 (2.09)	4.40 (3.52)	0.18 (0.68)
Fz3 (% BW)	82.68 (1.86)	**−14.24 (1.32)**	**−25.85 (1.32)**	**−35.91 (1.32)**	**−45.91 (1.32)**	−0.31 (1.32)	−4.10 (2.52)	0.29 (0.49)
Fz4 (% BW)	97.28 (1.64)	**−25.85 (1.19)**	**−36.46 (1.19)**	**−46.69 (1.19)**	**−57.19 (1.19)**	−0.67 (1.19)	−1.59 (2.21)	0.22 (0.43)

Estimated intercepts and mean differences to baseline for unloading, mean difference to female population for gender, and mean difference to mean BMI levels of the spatio-temporal, kinematic and kinetic LMMs as reported by the lmerTest package. Values in brackets specify averaged model standard errors of the coefficients. Bold coefficients correspond to significant mean differences to baseline from post-hoc testing

#### Descent

Only velocity (χ^2^(5) = 120.83, *p* = 5.44e-23) was affected by unloading while the other parameters showed no change. Post-hoc tests confirm that velocity is reduced from baseline at all levels of BWS except for the post measurement (Table 1).

### Kinematics

#### Ascent

**Fig. 3:**
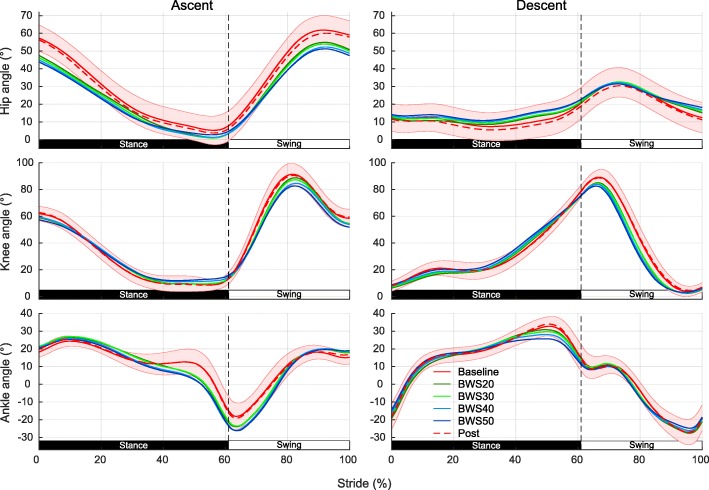
Lower limb joint angle trajectories during stair ascent and descent under various levels of unloading. Average hip, knee and ankle joint angles over all subjects for a single gait cycle during stair ascent and descent. Different line colors correspond to investigated BWS levels and shaded area to one standard deviation from mean baseline angle

Hip joint angle shortly before and after foot strike and ankle angle around foot off show the largest deviations especially at high unloading (Fig. 3). Overall, trajectory shapes remain largely conserved throughout unloading. LMM analysis of joint ROMs confirm that unloading has a significant effect on hip (χ^2^(5) = 63.85, *p* = 3.29e-11), knee (χ^2^(5) = 90.92, *p* = 9.04e-17), and ankle ROM (χ^2^(5) = 96.08, *p* = 7.78e-18). Compared to baseline, post-hoc tests show a reduction in hip and knee ROM and increase of ankle ROM at all unloading levels, while ROMs of post measurements do not differ (Table 1).

#### Descent

Changes are more subtle than for ascent and can be mainly seen in peak ankle flexion with substantial unloading while the overall joint trajectories stay quite similar to the baseline trajectory (Fig. 3). LMMs of joint ROMs nevertheless show, that unloading affects all joints with hip (χ^2^(5) = 69.08, *p* = 3.03e-12), knee (χ^2^(5) = 51.60, *p* = 9.78e-09), and ankle (χ^2^(5) = 104.87, *p* = 1.19e-19) being significantly affected. Post-hoc tests reveal that stair negotiation ROMs differ from baseline for all unloading levels apart from the 20% BWS condition of the ankle and the post conditions of all joints (Table 1). A difference between ascent and descent was that hip and ankle ROMs show for both directions a reduction with unloading while ankle ROMs are increased during ascent and decreased during descent. Male study participants show in addition a reduced ankle ROM of around 8–10° degrees compared to female participants during all conditions and stair negotiation directions.

### Kinetics

#### Ascent

**Fig. 4:**
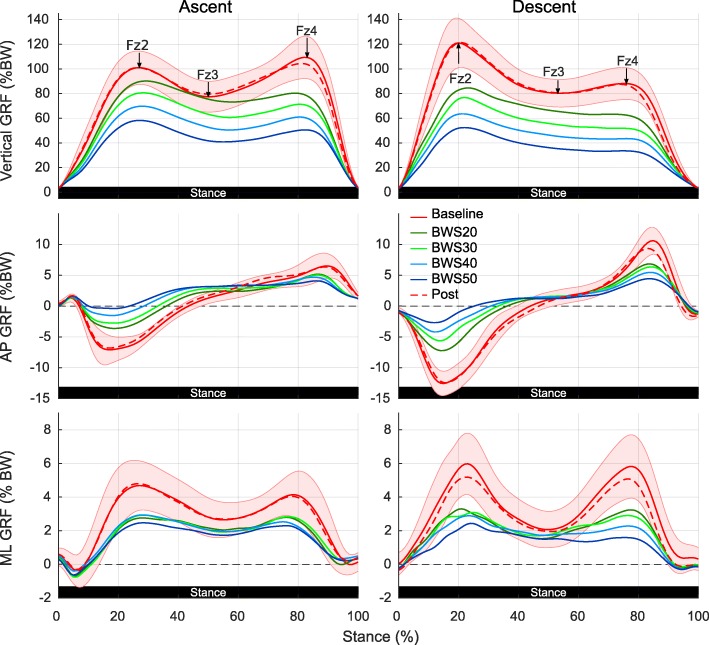
Ground reaction forces during stair ascent and descent under various levels of unloading. Average vertical, anteroposterior and mediolateral GRFs over all subjects for a single gait cycle during stair ascent and descent. Different line colors correspond to investigated BWS levels and shaded area to one standard deviation from mean baseline GRFs. Black arrows specify average vertical GRF peak (Fz2, Fz4) and plateau (Fz3) locations for the baseline condition and black dotted line visualizes the zero-force level

Vertical GRFs show a large force reduction for stair ascent which corresponds approximately with the level of unloading (Fig. 4). Anteroposterior (AP) GRFs show also large reductions with the first breaking peak (negative reaction force) being stronger affected than the second propulsion peak (positive reaction force) (Fig. 4). The breaking phase shortens relative to the propulsion phase. Likewise, mediolateral (ML) GRFs are reduced but different unloading levels show similar reductions (Fig. 4). LMM analysis confirms that Fz2 peak values are significantly influenced by unloading (χ^2^(5) = 300.05, *p* = 3.33e-61), as well as Fz3 plateaus (χ^2^(5) = 258.20, *p* = 2.87e-52) and Fz4 peaks (χ^2^(5) = 324.25, *p* = 2.14e-66). Post-hoc tests confirm that all levels of unloading differ from baseline and that the Fz4 peaks are reduced stronger than the Fz2 peaks (Table 1).

#### Descent

In line with stair ascent, vertical GRFs show large reductions corresponding to the unloading level with flattened GRF peaks (Fig. 4). In AP direction, both propulsive and braking peaks are reduced while duration of the propulsive phase is also relatively reduced (Fig. 4). ML GRFs are likewise smaller and show a nice gradual decrease related to unloading level (Fig. 4). LMMs reveal a significant effect of unloading on Fz2 peaks (χ^2^(5) = 290.71, *p* = 3.29e-59), Fz3 plateaus (χ^2^(5) = 286.73, *p* = 2.28e-58) and Fz4 peaks (χ^2^(5) = 351.09, *p* = 3.67e-72). Post-hoc tests again show significant the differences between unloading levels from baseline. Contrary to ascending, the vertical Fz2 peaks are stronger affected than the Fz4 peaks (Table 1).

### Myoelectric activity

#### Ascent

##### EMG envelopes

**Fig. 5:**
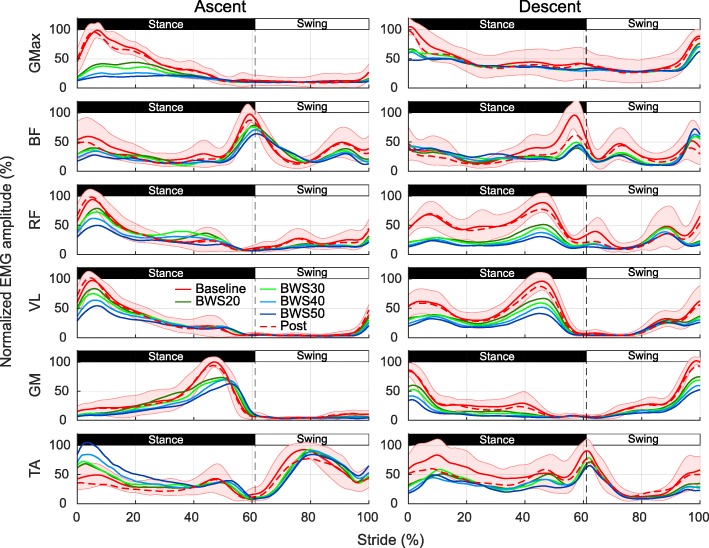
Surface EMG activity during stair ascent and descent under various levels of unloading. Averaged surface EMG activities over all subjects for a single gait cycle during stair ascent and descent. Different line colors correspond to investigated BWS levels and shaded area to one standard deviation from mean baseline EMG activity. Abbreviations: GMax, gluteus maximus; BF, biceps femoris; RF, rectus femoris; VL, vastus lateralis; GM, gastrocnemius medialis; TA, tibialis anterior

Apart from TA activity, all envelopes show reductions in myoelectric activity during peak myoelectric activity while being unloaded. For these muscles, BWS levels do not introduce any substantial compensatory activation patterns compared to baseline condition without unloading. Higher unloading levels result in the largest reductions while the post condition envelope remains highly similar compared to the baseline envelope. TA activity on the other hand shows an increase at the beginning of stance phase that scales positively with the amount of unloading. Effects of unloading on swing phase are less prominent than on stance phase (Fig. 5).

##### LMMs of myoelectric activity

LRTs confirm a general, significant effect of unloading on all muscle activities (GMax: χ^2^(5) = 184.88, *p* = 1.41e-36; BF: χ^2^(5) = 67.66, *p* = 5.65e-12; RF χ^2^(5) = 44.55, *p* = 2.51e-7; VL: χ^2^(5) = 99.34, *p* = 1.68e-18; GM: χ^2^(5) = 17.60, *p* = 3.49e-2; TA: χ^2^(5) = 23.77, *p* = 2.65e-3). For GMax and TA an interaction effect is found between unloading and gait phase (GMax: χ^2^(5) = 62.61, *p* = 1.26e-10; TA: χ^2^(5) = 25.27, *p* = 4.21e-03), which implies that swing and stance phase for these muscles are differently affected by unloading. In agreement with the envelope patterns, post-hoc tests reveal significant RMS EMG activity reduction for all muscles apart from GM at 40% BWS and TA during all unloading conditions (Table 2). For GMax stance phase and BF, RF and VL stance and swing phase reductions increase with higher unloading. GMax swing phase and GM stance and swing phase reductions do not scale strongly with unloading. TA on the other hand is the only muscle that shows largely increased stance phase activity at 40 and 50% BWS. Unloading has however no effect on TA’s swing phase activity. Post condition measurement activities do not differ significantly from baseline activity indicating that no obvious fatigue or adaptation effect seems to be present (Table 2).

**Table 2:** LMM mean difference estimates for lower limb myoelectric activity parameters

		Intercept (Base)	ΔBWS20	ΔBWS30	ΔBWS40	ΔBWS50	ΔPost	Δmale	ΔBMI
Ascent									
GMax (%)	Stance ΔSwing	103.01 (4.72) 3.16 (4.49)	**−43.28 (4.49)** **−22.04 (4.49)**	**−50.86 (4.49)** **− 22.44 (4.49)**	**−62.73 (4.49)** **− 24.12 (4.49)**	**−66.63 (4.49)** **− 26.10 (4.49)**	−3.38 (4.57) 1.67 (4.57)	−5.00 (5.53)	0.76 (1.07)
BF (%)	Stance ΔSwing	115.37 (8.00)	**− 22.18 (5.84)**	**− 21.70 (5.84)**	**−32.67 (5.84)**	**− 44.63 (5.84)**	−6.57 (5.84)	0.10 (10.47)	0.29 (2.03)
		**−17.77(3.37)**							
RF (%)	Stance ΔSwing	95.54 (5.72)	**− 17.05 (4.69)**	**−19.79 (4.69)**	**−23.54 (4.69)**	**−29.08 (4.69)**	−9.87 (4.69)	3.38 (7.11)	1.61 (1.38)
		**13.43 (2.71)**							
VL (%)	Stance ΔSwing	100.43 (5.50) −1.23 (2.63)	**−18.71 (4.56)**	**−24.82 (4.56)**	**−31.18 (4.56)**	**−40.99 (4.56)**	−1.80 (4.56)	12.08 (6.80)	0.26 (1.32)
GM (%)	Stance ΔSwing	87.44 (7.49)	**−18.70 (7.27)**	**−19.94 (7.27)**	− 15.16 (7.27)	**−21.64 (7.27)**	−1.00 (7.27)	17.76 (8.32)	0.98 (1.62)
		**17.22 (4.20)**							
TA (%)	Stance ΔSwing	114.44 (15.02) −7.67 (14.93)	22.58 (14.93) −6.55 (14.93)	35.35 (14.93) −4.75 (14.93)	**52.77 (14.93)**	**78.69 (14.93)**	−13.79 (14.93) −13.53 (14.93)	−9.48 (16.95)	0.92 (3.29)
					−6.58 (14.93)	−6.88 (14.93)			
Descent									
GMax (%)	Stance ΔSwing	110.18 (5.52) −0.52 (2.44)	**−27.88 (4.14)**	**−27.60 (4.14)**	**−26.04 (4.31)**	**−36.30 (4.22)**	−8.56 (4.22)	−9.87 (6.94)	0.33 (1.32)
BF (%)	Stance ΔSwing	102.06 (6.16) 7.66 (6.33)	**−49.30 (6.33)** **−37.28 (6.33)**	**−54.48 (6.33)** **− 37.89 (6.33)**	**−62.85 (6.33)** **− 46.37 (6.33)**	**−67.80 (6.33)** **− 52.72 (6.33)**	−5.44 (6.33)	− 0.47 (6.76)	0.01 (1.31)
							**−30.05 (6.33)**		
RF (%)	Stance ΔSwing	106.11 (7.23)	**−35.13 (6.01)**	**−22.54 (6.01)**	**−26.61 (6.12)**	**− 24.13 (6.01)**	**−23.75 (6.12)**	9.89 (8.94)	−3.43 (1.73)
		**−9.42 (3.50)**							
VL (%)	Stance ΔSwing	96.83 (3.43)	**−26.98 (3.33)**	**−33.68 (3.33)**	**− 39.07 (3.33)**	**−44.93 (3.33)**	**−10.84 (3.33)**	6.24 (3.81)	− 2.10 (0.74)
		**5.08 (1.92)**							
GM (%)	Stance ΔSwing	99.21 (2.79) 2.71 (1.61)	**−28.03 (2.80)**	**−35.55 (2.80)**	**−46.53 (2.80)**	**−53.39 (2.80)**	−5.82 (2.80)	2.15 (3.01)	0.40 (0.59)
TA (%)	Stance ΔSwing	109.84 (7.13) −2.58 (3.25)	**−27.26 (5.64)**	**− 25.15 (5.64)**	**−34.54 (5.64)**	**−37.17 (5.64)**	**−14.80 (5.64)**	1.91 (9.02)	1.90 (1.75)

Estimated intercepts and mean differences to baseline for unloading, mean difference to female population for gender, and mean difference to mean BMI levels of the myoelectric activity LMMs as reported by the lmerTest package. Values in brackets specify averaged model standard errors of the coefficients. Bold coefficients correspond to significant mean differences to baseline from post-hoc testing. Abbreviations: *GMax* Gluteus maximus, *BF* Biceps femoris, *RF* Rectus femoris, *VL* Vastus lateralis, *GM* Gastrocnemius medialis, *TA* Tibialis anterior

#### Descent

##### EMG envelopes

With unloading, all muscles show again distinct reductions in EMG envelope activity including TA. Largest reductions coincide with peak myoelectric activations and RF, VL, GM as well as TA show a general reduction over the whole stance phase. Swing phase activity is mainly affected right before the timing of foot strike with a visible reduction in all muscles apart from BF. No compensatory activation patterns are present in all EMG unloading patterns compared to baseline condition. Activity reduction also scale with the level of unloading and the post condition envelope shows a high resemblance to baseline activity.

##### LMMs of myoelectric activity

As for stair ascent, LRTs of the myoelectric activity models also show significant influence of unloading on all muscle activities (GMax: χ^2^(5) = 84.54, *p* = 1.88e-15; BF: χ^2^(5) = 155.57, *p* = 2.35e-30; RF: χ^2^(5) = 35.52, *p* = 1.54e-5; VL: χ^2^(5) = 170.59, *p* = 1.53e-33; GM: χ^2^(5) = 274.08, *p* = 1.15e-55; TA: χ^2^(5) = 53.52, *p* = 4.21e-9). Only BF shows a significant interaction between unloading and gait phase (χ^2^(5) = 31.94, *p* = 2.14e-04) while all other muscles have comparable reductions for stance and swing phases. Post-hoc tests comparing unloading conditions to baseline confirm reductions of stance and swing activities during all unloading conditions. For BF, VL, GM, and TA a scaling of the reduction with increasing unloading can be observed. BF in addition shows a stronger reduction with unloading during stance compared to swing phase. Differently than during stair ascent BF (only swing phase), RF, VL and TA show significant activity reductions even for the post condition. These reductions are however smaller or in case of RF at least not larger than the smallest reductions during unloading conditions.

## Discussion

In this study, effects of BWS on spatio-temporal, kinematic and kinetic parameters as well as lower leg muscle activities were investigated in a group of 17 able-bodied participants while ascending and descending stairs. Stair negotiation without unloading was compared to performance with 20 to 50% BWS. During ascent and descent, unloading resulted in statistically significant alterations of all myoelectric activities, kinematic, and kinetic parameters. Spatio-temporal parameters mainly remained unaffected. The observed alterations are global changes and do not represent non-physiological patterns. Only TA activity during ascent showed a slightly different pattern during unloading. BWS stair negotiation therewith reflects previous findings from overground walking which state that unloading does not strongly perturb movement performance [19, 20]. This conservation of kinematic, kinetic and myoelectric activation patterns is one of the main concepts underlaying task-specific, locomotor training and has been advocated to be a key requirement for successful rehabilitation [4, 39].

Although kinematic patterns remain similar, substantial unloading (40 to 50% BWS) still introduces sizeable reductions in ROM for ascent and descent and compensatory TA activity for ascent in able-bodied participants. Similar but less prominent changes have been found for ROMs and other muscles during overground [19, 20] or treadmill walking with BWS [19]. The observable changes are either due to adaptations of motor patterns [40], or an inevitable consequence of direct mechanical and passive interactions of unloading. While these alterations are not ideal, they are not so prominent that we would advise refraining from training stair negotiation in patients due to safety concerns. Compared with level walking, stair negotiation has higher neuromuscular complexity and greater ROM requirements. Therefore, it is not surprising that the responses to unloading are exacerbated. As demonstrated in level walking, patient populations can display specific response profiles to unloading which deviate from able-bodied responses – this potentially offers a unique window on recovery and reasons for recovery limitations [21].

The following sections discuss for each subgroup of parameters possible sources and magnitude of the deviations from baseline measurement.

### Spatio-temporal parameters

The clearest effect of unloading on spatio-temporal parameters is observable on walking speed measured as center of mass (COM) velocity. While walking speed during the baseline condition is comparable to previously conducted stair studies [7], a significant reduction with increasing unloading is found. While a speed reduction is expected for stair descent due to a damping of the downward motion (BWS acts as a resistance that slows motion towards earth), an increase could be expected for stair ascent due to the acceleration of upward motion. Reasons for a reduction in both directions can also be that unloading reduces the vertical breaking energy which can under baseline condition be stored and partially reused for propulsion, hence the reduction in propulsion under unloading. This effect is well-known from experiments with parabola flights [40, 41] or simulated reduced gravity as with BWS [40, 42]. In patients this reduction can be masked by an increased walking speed due to the enabling properties of BWS systems [42]. Another reason for a reduction in walking speed are small increases in resistive interaction forces between BWS and its user with increasing unloading [26] or difficulties in trunk flexion due to vertical unloading which is necessary to generate forward propulsion [43]. Recent clinical studies for overground walking in patients with spinal cord injury however showed that the reduction in speed from BWS can be overcome with providing appropriate forward forces besides vertical BWS [22].

During stair ascent, a reduction of step width at low BWS levels (20–30%) is present which might indicate increased stability through unloading [44]. This stabilizing effect might be lost with higher unloading due to decreases in the gravitational moment about the stance limb [45]. Stair descent seems to be less affected by unloading because step width effects became insignificant with the multiple comparison corrections.

During ascent single support phase duration increased which is also known from overground walking with BWS [20, 46, 47]. One reason might be that BWS provides external stabilization [44, 45] allowing participants to spend more time on a single leg without expending larger neuromuscular efforts [48–50]. Patients with balance problems could therefore profit through BWS from a reduced fall risk. A second reason is that BWS applied via a harness reduces load on the stance leg while the swing leg remains largely unsupported [51]. These differences in leg dynamics can then result in temporal alterations. Compared to overground walking however, other temporal parameters remained unaffected. One reason for this might be that – in contrast to overground walking – the step length is fixed by the stair tread depth so that spatio-temporal adaptation possibilities are reduced.

### Gait kinematics

Comparison of baseline kinematics with other studies in able-bodied subjects during stair negotiation shows mostly comparable joint ROMs for similar stair dimensions [7, 31, 32]. Apart from different stair dimensions, differences in ROMs might arise from differences in marker placement or study population demographics. In this study, which is the first to investigate unloading effects on stair negotiation, a general reduction in hip and knee ROMs during ascent and descent is seen while ankle ROM increases during ascent and decreases during descent. These changes in ROM can be attributed to the extending effect unloading has on the joints and are in line with, albeit smaller, ROM reduction for BWS overground and treadmill walking [19, 20]. Differences in ankle ROM between stair ascent and descent can be explained the same way. During ascent ankle dorsiflexion increases because BWS is lifting the body upwards while ankle plantarflexion decreases at the end of stance phase due to a more upright posture. Post-hoc tests show that effects seem to scale with increased BWS. Although walking speed could not be strictly kept constant over all investigated conditions, effect of speed on joint angles as shown by Lewis et al. [52] are much smaller than the here observed effects so that these changes can be indeed in large part attributed to BWS increases. For level ground walking the effect of speed on joint trajectories [53] is much more pronounced than for stair negotiation, which might be a direct consequence of the fixed step length during stair walking.

### Ground reaction forces

In line with results from Barela et al. for overground walking [54], increasing BWS decreases ground reaction forces also during stair negotiation. Peak and plateau values become closer with larger BWS. Ascending vertical GRFs show normally a higher second peak due to the larger impact of the push-off compared to weight acceptance phase [32]. With all levels of unloading however, the push-off peak (Fz4) becomes smaller than the weight acceptance peak (Fz2) which shows that BWS takes over a large part of vertical COM transfer. During stair descent, push-off/lowering peaks are on the other hand usually smaller than weight acceptance peaks [32]. This difference remains even under BW unloading and a continuous decrease in GRF is the consequence between these two peaks instead while the intermediate plateau vanishes.

AP and ML GRFs have not been quantified using parameters but from the continuous diagrams it becomes clear that for AP GRFs breaking impulses at the beginning of stance phase are strongly reduced while propulsion impulses have a reduced peak but are extended in their relative percentage over stance phase duration. In descending direction AP and ML GRFs are also larger than in ascending direction which probably indicates a greater balance demand [55]. With all levels of unloading these differences become smaller for ML GRFs.

### Myoelectric activity

McFadyen and Winter were the first authors to offer a complete biomechanical analysis of normative stair ascent and descent including surface electromyograms of all major leg muscles [33]. The myoelectric activities we observed in our participants during baseline condition match their observed EMG envelope patterns closely. Even the shape of surface EMG envelopes during substantial unloading remained very similar to the natural EMG pattern but peak activations were however flattened showing that lower myoelectric activity is required for ascending and descending stairs. The amplitude reduction in all muscles increased with larger unloading apart from GM and TA during ascent as well as RF during descent. Large, relative amplitude reductions during ascent were especially present in GMax, BF and VL activity. VL and GMax are known to both contribute to vertical propulsion of the body through knee and hip extension and transfer of power from the contralateral leg for GMax [33, 56]. GMax is also believed to contribute to COM forward propulsion during early stance while BF might create forward propulsion during late stance [33, 56]. A reduction in these muscles is therefore highly likely a combination from the vertical assistance of the BWS and the reduced speed with higher unloading. TA on the other hand showed a highly variable but increased stance activity under 40–50% BWS during ascent. It is the only muscle with increased activity showing compensatory activity. We hypothesize that subjects increased TA activity to compensate and lean forward to help with forward transfer of the center of mass during some of the stair steps which also explains the large range in amplitude values. With BWS this forward shifting might be hindered due to erection of the whole body which might then play a part in the lower velocities that were observed with increasing BWS. Awai et al. reported similar compensatory activation in BF activity which they linked to compensation of forward propulsion that decreased due to GM activity reduction [20]. However, reduction of EMG amplitudes was not only restricted to the stance phase but also showed a reduction of activity for most peaks during swing phase. Mechanically, BWS should however mainly affect the stance leg leaving the swing leg unaffected [51]. TA, the muscle with the clearest swing activity during ascent and responsible for appropriate toe clearance, then also showed no reduction in swing EMG amplitudes with unloading. An explanation why other muscles show a similar swing phase amplitude reduction compared to stance phase might be a change in their preparatory activity before foot strike [57–59]. Due to the familiarization to the BWS conditions before the measurements, participants expect lower upcoming impact forces and reduce their muscular preactivation accordingly. The reduction in the swing phase therefore also becomes greatest at the very end. The impact during stair descent is naturally larger and all extensors are involved in slowly accepting the weight when landing which might be why during descent all muscles show an amplitude reduction during both stance and swing phases. For stair ascent, post condition amplitudes are not significantly different from baseline amplitudes while for descent even during post condition small significant amplitude reductions persist. Adaptation effects to the reduced gravity might therefore indeed take place in this direction so that participants get used to the slowed down lowering and rely more on passive structural mechanics than active breaking. However, kinematic changes are not present so this phenomenon will require additional investigations. Compared with overground walking [19, 20], the effects of unloading on muscular activity is much larger in stair ascent and descent which might stem from the fact that during stair negotiation larger moments need to be provided from the muscles to generate a large vertical translation of the body which is directly supported by BWS.

The goal of body weight unloading in rehabilitation is to facilitate practice of gait-related activities so that patients who would not be able to train a task, or train only for a limited amount of time, can train longer and start earlier with training. Both early start of rehabilitation, additional practice and higher training intensity are an integral component of today’s clinical practice and have been shown to be paramount for optimal functional recovery [10, 11, 60, 61]. The arguably largest challenge of negotiating stairs for patients is the high muscular demands compared to overground walking. Although surface EMG amplitude cannot be related to muscle force in a simple manner [62], the reduction in myoelectric activity presented in this study in able-bodied subjects indicates a reduction in neuromuscular demand that is probably related to a facilitation of the task. We therefore hypothesize that negotiating stairs with unloading should be achievable even with reduced physical strength as it is the case for many patients with neurologic injuries while no abnormal compensatory activity patterns are introduced from the BWS. Furthermore, the safety provided through the body-weight support should enable these patients and others with for example impaired lower limb coordination or balance impairments to train stair negotiation patterns and step clearance without fear of falling and injuring oneself. These hypotheses must however in a next step be investigated with each specific patient population.

### Outlook

One of the limitations of this study is that it was not possible to investigated unloading effects down to 10% BWS due to slow upward acceleration of the BWS system’s end-effector at this unloading level during ascent. As a result, subjects would collide with the robotic end-effector at their self-selected speed. In a slow walking patient population however even 10% BWS can work so the limitation only applies for faster walking speeds. In addition, reductions in walking speed, joint ROMs at substantial unloading and compensatory activation of TA could maybe be lessened by adding forward forces to the vertical BWS. A study with overground BWS in patients showed that tailored forward assistance can improve gait pattern and walking velocities to a large degree [22]. A similar approach could be applied to stair negotiation so that the walking speed can be kept close to the self-selected speed. Additionally, modulating the vertical BWS or forward forces based on gait phase events or trunk motion could reduce observed alterations of BWS during stair negotiation even more and might be beneficial for patients with unilateral deficits [63]. This could enable a wide range of patients to train stair negotiation in a physiological way without fear of falls. As a next step, measurements with patients with various gait dysfunctions are required to show how well different patients can harness the permissive environment created by the BWS system. It has to be investigated if these patients show similar adaptations to BWS compared to able-bodied stair walkers or other patient groups and if the BWS training leads in these patients to meaningful functional improvements over time.

## Conclusions

In this study, we investigate the effects of unloading on kinematic patterns, myoelectric activity, and ground reaction forces during stair negotiation in able-bodied subjects. Our results show, that in line with studies on BWS during treadmill and overground walking, BWS during stair negotiation as well fulfills its role of reducing participants’ body weight without compromising kinematic and muscular patterns greatly up to 30% BWS. Beyond 30% BWS, ROMs are systematically reduced as an inherent consequence of substantial unloading and compensatory TA activity was detected. Therefore, our data implies that up to 30% BWS should be applicable in patient trainings without altering the movement patterns of the real-world task. If future investigations show the same benefit of BWS to reduce neuromuscular demands and ground reaction forces while complying with key locomotor retraining principles and providing a safe and permissive environment in patients, BWS can be key to stair training early in the rehabilitation treatment plan.

## Supplementary information

**Additional file 1.** This file includes LRT statistics for the parameters which were not significantly affected by unloading. It additionally includes detailed post-hoc test statistics for comparisons between baseline and unloading conditions for all investigated parameters.

## Data Availability

The datasets used and/or analyzed during the current study are available from the corresponding author on reasonable request.

## Abbreviations

AP: Anteroposterior
BF: Biceps Femoris
BW: Body weight
BWS: Body weight support
COM: Center of mass
EMG: Electromyographic
GM: Gastrocnemius Medialis
GMax: Gluteus Maximus
LMM: Linear mixed (effects) model
LRT: Likelihood Ratio Tests
ML: Mediolateral
RF: Rectus Femoris
RMS: Root-mean square
ROM: Range of motion
TA: Tibialis Anterior
VL: Vastus Lateralis
