# Supplementary material for: Robotic body weight support enables safe stair negotiation in compliance with basic locomotor principles
Source: J Neuroeng Rehabil. 2019 Dec 23;16:157. doi: 10.1186/s12984-019-0631-8 (PMC6929285; doi:10.1186/s12984-019-0631-8)
Supplement: Supplementary file 1 — Additional file 1. This file includes LRT statistics for the parameters which were not significantly affected by unloading. It additionally includes detailed post-hoc test statistics for comparisons between baseline and unloading conditions for all investigated parameters. [file 12984_2019_631_MOESM1_ESM.docx]

***SUPPLEMENTARY STATISTICS***

**Likelihood Ratio Tests**

*Ascent*

Non significantly affected parameters by unloading had the following chi-square distributions: Stance duration (χ^2^(5) = 4.17, p = 1) swing duration (χ^2^(5) = 3.86, p = 1) and double support duration (χ^2^(5) = 6.22, p = 1).

*Descent*

Non significantly affected parameters by unloading had the following chi-square distributions: Step width (χ^2^(5) = 13.79, p = 1.36e-1), stance duration (χ^2^(5) = 4.17, p = 1), swing duration (χ^2^(5) = 3.86, p = 1), single support duration (χ^2^(5) = 4.93, p = 1) and double support duration (χ^2^(5) = 3.44, p = 1).

**Post-hoc Tests**

*Ascent*

| Velocity | | | | | |
| --- | --- | --- | --- | --- | --- |
| Condition | Estimate | Std. Error | z-value | p-value | significance |
| ΔBWS20 | -0.069910 | 0.008905 | -7.851 | <0.001 | *** |
| ΔBWS30 | -0.074117 | 0.008905 | -8.323 | <0.001 | *** |
| ΔBWS40 | -0.089684 | 0.008905 | -10.071 | <0.001 | *** |
| ΔBWS50 | -0.110586 | 0.008905 | -12.419 | <0.001 | *** |
| ΔPost | -0.013779 | 0.008905 | -1.547 | 0.390 |  |

| Step Width | | | | | |
| --- | --- | --- | --- | --- | --- |
| Condition | Estimate | Std. Error | z-value | p-value | significance |
| ΔBWS20 | -0.021947 | 0.005202 | -4.219 | <0.001 | *** |
| ΔBWS30 | -0.023317 | 0.005202 | -4.482 | <0.001 | *** |
| ΔBWS40 | -0.007244 | 0.005202 | -1.392 | 0.493 |  |
| ΔBWS50 | -0.003778 | 0.005202 | -0.726 | 0.923 |  |
| ΔPost | -0.008326 | 0.005202 | -1.600 | 0.357 |  |

| Single Support | | | | | |
| --- | --- | --- | --- | --- | --- |
| Condition | Estimate | Std. Error | z-value | p-value | significance |
| ΔBWS20 | 0.014731 | 0.005019 | 2.935 | 0.01489 | * |
| ΔBWS30 | 0.013216 | 0.005019 | 2.633 | 0.03607 | * |
| ΔBWS40 | 0.015787 | 0.005019 | 3.145 | 0.00734 | ** |
| ΔBWS50 | 0.017070 | 0.005019 | 3.401 | 0.00314 | ** |
| ΔPost | 0.006321 | 0.005019 | 1.259 | 0.58821 |  |

| Hip ROM | | | | | |
| --- | --- | --- | --- | --- | --- |
| Condition | Estimate | Std. Error | z-value | p-value | significance |
| ΔBWS20 | -2.8693 | 1.0466 | -2.742 | 0.02662 | * |
| ΔBWS30 | -3.6763 | 1.0466 | -3.513 | 0.00216 | ** |
| ΔBWS40 | -5.7089 | 1.0466 | -5.455 | < 0.001 | *** |
| ΔBWS50 | -8.0935 | 1.0466 | -7.733 | < 0.001 | *** |
| ΔPost | -0.2722 | 1.0466 | -0.260 | 0.99919 |  |

| Knee ROM | | | | | |
| --- | --- | --- | --- | --- | --- |
| Condition | Estimate | Std. Error | z-value | p-value | significance |
| ΔBWS20 | -3.0449 | 1.1766 | -2.588 | 0.0408 | * |
| ΔBWS30 | -4.5949 | 1.1766 | -3.905 | <0.001 | *** |
| ΔBWS40 | -8.8369 | 1.1766 | -7.510 | <0.001 | *** |
| ΔBWS50 | -11.4340 | 1.1766 | -9.718 | <0.001 | *** |
| ΔPost | -0.3742 | 1.1766 | -0.318 | 0.9979 |  |

| Ankle ROM | | | | | |
| --- | --- | --- | --- | --- | --- |
| Condition | Estimate | Std. Error | z-value | p-value | significance |
| ΔBWS20 | 7.692 | 1.028 | 7.484 | <0.001 | *** |
| ΔBWS30 | 7.782 | 1.028 | 7.571 | <0.001 | *** |
| ΔBWS40 | 9.713 | 1.028 | 9.451 | <0.001 | *** |
| ΔBWS50 | 9.357 | 1.028 | 9.104 | <0.001 | *** |
| ΔPost | 1.363 | 1.028 | 1.326 | 0.54 |  |

| Fz2 | | | | | |
| --- | --- | --- | --- | --- | --- |
| Condition | Estimate | Std. Error | z-value | p-value | significance |
| ΔBWS20 | -10.4313 | 1.1132 | -9.371 | <1e-05 | *** |
| ΔBWS30 | -20.5412 | 1.1132 | -18.453 | <1e-05 | *** |
| ΔBWS40 | -31.7014 | 1.1132 | -28.479 | <1e-05 | *** |
| ΔBWS50 | -43.1334 | 1.1132 | -38.749 | <1e-05 | *** |
| ΔPost | -0.6003 | 1.1132 | -0.539 | 0.977 |  |

| Fz3 | | | | | |
| --- | --- | --- | --- | --- | --- |
| Condition | Estimate | Std. Error | z-value | p-value | significance |
| ΔBWS20 | -4.286 | 1.402 | -3.057 | 0.0102 | * |
| ΔBWS30 | -16.550 | 1.402 | -11.804 | <0.001 | *** |
| ΔBWS40 | -27.205 | 1.402 | -19.403 | <0.001 | *** |
| ΔBWS50 | -37.583 | 1.402 | -26.805 | <0.001 | *** |
| ΔPost | 3.191 | 1.402 | 2.276 | 0.0904 |  |

| Fz4 | | | | | |
| --- | --- | --- | --- | --- | --- |
| Condition | Estimate | Std. Error | z-value | p-value | significance |
| ΔBWS20 | -28.520 | 1.490 | -19.143 | <1e-04 | *** |
| ΔBWS30 | -37.899 | 1.490 | -25.438 | <1e-04 | *** |
| ΔBWS40 | -48.327 | 1.490 | -32.437 | <1e-04 | *** |
| ΔBWS50 | -59.541 | 1.490 | -39.965 | <1e-04 | *** |
| ΔPost | -1.472 | 1.490 | -0.988 | 0.782 |  |

| GMax | | | | | |
| --- | --- | --- | --- | --- | --- |
| Condition | Estimate | Std. Error | z-value | p-value | significance |
| Stance |  |  |  |  |  |
| ΔBWS20 | -43.280 | 4.492 | -9.635 | < 1e-06 | *** |
| ΔBWS30 | -50.862 | 4.492 | -11.323 | < 1e-06 | *** |
| ΔBWS40 | -62.732 | 4.492 | -13.966 | < 1e-06 | *** |
| ΔBWS50 | -66.633 | 4.492 | -14.834 | < 1e-06 | *** |
| ΔPost | -3.383 | 4.567 | -0.741 | 0.993 |  |
| Swing |  |  |  |  |  |
| ΔBWS20 | -22.035 | 4.492 | -4.906 | 8.70e-06 | *** |
| ΔBWS30 | -22.442 | 4.492 | -4.996 | 5.03e-06 | *** |
| ΔBWS40 | -24.118 | 4.492 | -5.369 | < 1e-06 | *** |
| ΔBWS50 | -26.103 | 4.492 | -5.811 | < 1e-06 | *** |
| ΔPost | 1.671 | 4.567 | 0.366 | 1.000 |  |

| RF | | | | | |
| --- | --- | --- | --- | --- | --- |
| Condition | Estimate | Std. Error | z-value | p-value | significance |
| ΔBWS20 | -22.176 | 5.839 | -3.798 | <0.001 | *** |
| ΔBWS30 | -21.703 | 5.839 | -3.717 | <0.001 | *** |
| ΔBWS40 | -32.668 | 5.839 | -5.595 | <0.001 | *** |
| ΔBWS50 | -44.634 | 5.839 | -7.644 | <0.001 | *** |
| ΔPost | -6.572 | 5.839 | -1.125 | 0.686 |  |

| BF | | | | | |
| --- | --- | --- | --- | --- | --- |
| Condition | Estimate | Std. Error | z-value | p-value | significance |
| ΔBWS20 | -17.052 | 4.689 | -3.636 | 0.00119 | ** |
| ΔBWS30 | -19.794 | 4.689 | -4.221 | < 0.001 | *** |
| ΔBWS40 | -23.541 | 4.689 | -5.020 | < 0.001 | *** |
| ΔBWS50 | -29.084 | 4.689 | -6.202 | < 0.001 | *** |
| ΔPost | -9.865 | 4.689 | -2.104 | 0.13450 |  |

| VL | | | | | |
| --- | --- | --- | --- | --- | --- |
| Condition | Estimate | Std. Error | z-value | p-value | significance |
| ΔBWS20 | -18.710 | 4.563 | -4.100 | < 1e-04 | *** |
| ΔBWS30 | -24.815 | 4.563 | -5.438 | < 1e-04 | *** |
| ΔBWS40 | -31.179 | 4.563 | -6.833 | < 1e-04 | *** |
| ΔBWS50 | -40.992 | 4.563 | -8.984 | < 1e-04 | *** |
| ΔPost | -1.796 | 4.563 | -0.394 | 0.994277 |  |

| GM | | | | | |
| --- | --- | --- | --- | --- | --- |
| Condition | Estimate | Std. Error | z-value | p-value | significance |
| ΔBWS20 | -18.697 | 7.273 | -2.571 | 0.0427 | * |
| ΔBWS30 | -19.939 | 7.273 | -2.742 | 0.0265 | * |
| ΔBWS40 | -15.161 | 7.273 | -2.085 | 0.1403 |  |
| ΔBWS50 | -21.643 | 7.273 | -2.976 | 0.0132 | * |
| ΔPost | -1.000 | 7.273 | -0.138 | 1.0000 |  |

| TA | | | | | |
| --- | --- | --- | --- | --- | --- |
| Condition | Estimate | Std. Error | z-value | p-value | significance |
| Stance |  |  |  |  |  |
| ΔBWS20 | 22.581 | 14.932 | 1.512 | 0.65433 |  |
| ΔBWS30 | 35.350 | 14.932 | 2.367 | 0.13958 |  |
| ΔBWS40 | 52.772 | 14.932 | 3.534 | 0.00386 | ** |
| ΔBWS50 | 78.694 | 14.932 | 5.270 | < 1e-04 | *** |
| ΔPost | -13.790 | 14.932 | -0.924 | 0.96864 |  |
| Swing |  |  |  |  |  |
| ΔBWS20 | -6.553 | 14.932 | -0.439 | 0.99991 |  |
| ΔBWS30 | -4.746 | 14.932 | -0.318 | 1.00000 |  |
| ΔBWS40 | -6.580 | 14.932 | -0.441 | 0.99991 |  |
| ΔBWS50 | -6.882 | 14.932 | -0.461 | 0.99986 |  |
| ΔPost | -13.530 | 14.932 | -0.906 | 0.97224 |  |

*Descent*

| Velocity | | | | | |
| --- | --- | --- | --- | --- | --- |
| Condition | Estimate | Std. Error | z-value | p-value | significance |
| ΔBWS20 | -0.083664 | 0.009984 | -8.380 | <1e-04 | *** |
| ΔBWS30 | -0.087165 | 0.009984 | -8.731 | <1e-04 | *** |
| ΔBWS40 | -0.104693 | 0.009984 | -10.486 | <1e-04 | *** |
| ΔBWS50 | -0.121385 | 0.009984 | -12.158 | <1e-04 | *** |
| ΔPost | -0.012317 | 0.009984 | -1.234 | 0.607 |  |

| Hip ROM | | | | | |
| --- | --- | --- | --- | --- | --- |
| Condition | Estimate | Std. Error | z-value | p-value | significance |
| ΔBWS20 | -1.7161 | 0.5389 | -3.184 | 0.00656 | ** |
| ΔBWS30 | -1.7564 | 0.5389 | -3.259 | 0.00501 | ** |
| ΔBWS40 | -3.0151 | 0.5389 | -5.595 | < 0.001 | *** |
| ΔBWS50 | -4.4855 | 0.5389 | -8.323 | < 0.001 | *** |
| ΔPost | -0.1724 | 0.5389 | -0.320 | 0.99782 |  |

| Knee ROM | | | | | |
| --- | --- | --- | --- | --- | --- |
| Condition | Estimate | Std. Error | z-value | p-value | significance |
| ΔBWS20 | -2.9974 | 0.8181 | -3.664 | 0.00125 | ** |
| ΔBWS30 | -3.7299 | 0.8181 | -4.559 | < 0.001 | *** |
| ΔBWS40 | -4.1231 | 0.8181 | -5.040 | < 0.001 | *** |
| ΔBWS50 | -5.9086 | 0.8181 | -7.222 | < 0.001 | *** |
| ΔPost | -1.2391 | 0.8181 | -1.515 | 0.41048 |  |

| Ankle ROM | | | | | |
| --- | --- | --- | --- | --- | --- |
| Condition | Estimate | Std. Error | z-value | p-value | significance |
| ΔBWS20 | -1.4454 | 0.8057 | -1.794 | 0.253 |  |
| ΔBWS30 | -4.0261 | 0.8057 | -4.997 | <0.001 | *** |
| ΔBWS40 | -5.3979 | 0.8057 | -6.700 | <0.001 | *** |
| ΔBWS50 | -8.2745 | 0.8057 | -10.270 | <0.001 | *** |
| ΔPost | 1.1432 | 0.8057 | 1.419 | 0.474 |  |

| Fz2 | | | | | |
| --- | --- | --- | --- | --- | --- |
| Condition | Estimate | Std. Error | z-value | p-value | significance |
| ΔBWS20 | -37.579 | 2.086 | -18.014 | <1e-06 | *** |
| ΔBWS30 | -46.329 | 2.086 | -22.208 | <1e-06 | *** |
| ΔBWS40 | -59.400 | 2.086 | -28.474 | <1e-06 | *** |
| ΔBWS50 | -72.489 | 2.086 | -34.748 | <1e-06 | *** |
| ΔPost | -1.041 | 2.086 | -0.499 | 0.983 |  |

| Fz3 | | | | | |
| --- | --- | --- | --- | --- | --- |
| Condition | Estimate | Std. Error | z-value | p-value | significance |
| ΔBWS20 | -14.2353 | 1.3175 | -10.805 | <1e-08 | *** |
| ΔBWS30 | -25.8475 | 1.3175 | -19.618 | <1e-08 | *** |
| ΔBWS40 | -35.9127 | 1.3175 | -27.257 | <1e-08 | *** |
| ΔBWS50 | -45.9103 | 1.3175 | -34.846 | <1e-08 | *** |
| ΔPost | -0.3141 | 1.3175 | -0.238 | 0.999 |  |

| Fz4 | | | | | |
| --- | --- | --- | --- | --- | --- |
| Condition | Estimate | Std. Error | z-value | p-value | significance |
| ΔBWS20 | -25.8504 | 1.1847 | -21.821 | <1e-05 | *** |
| ΔBWS30 | -36.4629 | 1.1847 | -30.779 | <1e-05 | *** |
| ΔBWS40 | -46.6940 | 1.1847 | -39.415 | <1e-05 | *** |
| ΔBWS50 | -57.1931 | 1.1847 | -48.278 | <1e-05 | *** |
| ΔPost | -0.6731 | 1.1847 | -0.568 | 0.971 |  |

| GMax | | | | | |
| --- | --- | --- | --- | --- | --- |
| Condition | Estimate | Std. Error | z-value | p-value | significance |
| ΔBWS20 | -27.875 | 4.139 | -6.735 | <0.001 | *** |
| ΔBWS30 | -27.595 | 4.139 | -6.668 | <0.001 | *** |
| ΔBWS40 | -26.037 | 4.310 | -6.041 | <0.001 | *** |
| ΔBWS50 | -36.298 | 4.221 | -8.600 | <0.001 | *** |
| ΔPost | -8.557 | 4.219 | -2.028 | 0.16 |  |

| RF | | | | | |
| --- | --- | --- | --- | --- | --- |
| Condition | Estimate | Std. Error | z-value | p-value | significance |
| Stance |  |  |  |  |  |
| ΔBWS20 | -49.301 | 6.331 | -7.787 | < 1e-05 | *** |
| ΔBWS30 | -54.484 | 6.331 | -8.606 | < 1e-05 | *** |
| ΔBWS40 | -62.849 | 6.331 | -9.927 | < 1e-05 | *** |
| ΔBWS50 | -67.799 | 6.331 | -10.709 | < 1e-05 | *** |
| ΔPost | -5.435 | 6.331 | -0.858 | 0.981 |  |
| Swing |  |  |  |  |  |
| ΔBWS20 | -37.278 | 6.331 | -5.888 | < 1e-05 | *** |
| ΔBWS30 | -37.891 | 6.331 | -5.985 | < 1e-05 | *** |
| ΔBWS40 | -46.365 | 6.331 | -7.324 | < 1e-05 | *** |
| ΔBWS50 | -52.716 | 6.331 | -8.327 | < 1e-05 | *** |
| ΔPost | -30.045 | 6.331 | -4.746 | 1.99e-05 | *** |

| BF | | | | | |
| --- | --- | --- | --- | --- | --- |
| Condition | Estimate | Std. Error | z-value | p-value | significance |
| ΔBWS20 | -35.133 | 6.006 | -5.850 | < 1e-04 | *** |
| ΔBWS30 | -22.538 | 6.006 | -3.752 | 0.000779 | *** |
| ΔBWS40 | -26.609 | 6.117 | -4.350 | < 1e-04 | *** |
| ΔBWS50 | -24.129 | 6.006 | -4.018 | 0.000254 | *** |
| ΔPost | -23.750 | 6.117 | -3.883 | 0.000469 | *** |

| VL | | | | | |
| --- | --- | --- | --- | --- | --- |
| Condition | Estimate | Std. Error | z-value | p-value | significance |
| ΔBWS20 | -26.98 | 3.33 | -8.103 | < 0.001 | *** |
| ΔBWS30 | -33.68 | 3.33 | -10.115 | < 0.001 | *** |
| ΔBWS40 | -39.07 | 3.33 | -11.732 | < 0.001 | *** |
| ΔBWS50 | -44.93 | 3.33 | -13.494 | < 0.001 | *** |
| ΔPost | -10.84 | 3.33 | -3.254 | 0.00513 | ** |

| GM | | | | | |
| --- | --- | --- | --- | --- | --- |
| Condition | Estimate | Std. Error | z-value | p-value | significance |
| ΔBWS20 | -28.027 | 2.795 | -10.029 | <0.001 | *** |
| ΔBWS30 | -35.545 | 2.795 | -12.719 | <0.001 | *** |
| ΔBWS40 | -46.526 | 2.795 | -16.648 | <0.001 | *** |
| ΔBWS50 | -53.387 | 2.795 | -19.103 | <0.001 | *** |
| ΔPost | -5.816 | 2.795 | -2.081 | 0.141 |  |

| TA | | | | | |
| --- | --- | --- | --- | --- | --- |
| Condition | Estimate | Std. Error | z-value | p-value | significance |
| ΔBWS20 | -27.256 | 5.637 | -4.835 | <0.001 | *** |
| ΔBWS30 | -25.149 | 5.637 | -4.461 | <0.001 | *** |
| ΔBWS40 | -34.542 | 5.637 | -6.127 | <0.001 | *** |
| ΔBWS50 | -37.170 | 5.637 | -6.594 | <0.001 | *** |
| ΔPost | -14.796 | 5.637 | -2.625 | 0.0367 | * |
